# Pulsed electric field increases the extraction yield of extra virgin olive oil without loss of its biological properties

**DOI:** 10.3389/fnut.2022.1065543

**Published:** 2022-11-22

**Authors:** Roberto Martínez-Beamonte, Marina Ripalda, Tania Herrero-Continente, Cristina Barranquero, Alberto Dávalos, María Carmen López de las Hazas, Ignacio Álvarez-Lanzarote, Ana Cristina Sánchez-Gimeno, Javier Raso, Carmen Arnal, Joaquín C. Surra, Jesús Osada, María A. Navarro

**Affiliations:** ^1^Departamento de Bioquímica y Biología Molecular y Celular, Facultad de Veterinaria, Instituto de Investigación Sanitaria de Aragón, Universidad de Zaragoza, Zaragoza, Spain; ^2^Instituto Agroalimentario de Aragón, CITA-Universidad de Zaragoza, Zaragoza, Spain; ^3^CIBER de Fisiopatología de la Obesidad y Nutrición, Instituto de Salud Carlos III, Madrid, Spain; ^4^Laboratory of Epigenetics of Lipid Metabolism, Instituto Madrileño de Estudios Avanzados (IMDEA)-Alimentación, CEI UAM + CSIC, Madrid, Spain; ^5^Departamento de Producción Animal y Ciencia de los Alimentos, Facultad de Veterinaria, Universidad de Zaragoza, Zaragoza, Spain; ^6^Departamento de Patología Animal, Facultad de Veterinaria, Instituto de Investigación Sanitaria de Aragón, Universidad de Zaragoza, Zaragoza, Spain; ^7^Departamento de Producción Animal y Ciencia de los Alimentos, Escuela Politécnica Superior de Huesca, Instituto de Investigación Sanitaria de Aragón, Universidad de Zaragoza, Zaragoza, Spain

**Keywords:** extra virgin olive oil, Empeltre, microRNA, pulsed electric field, hepatic steatosis, atherosclerosis, phytosterols

## Abstract

**Introduction:**

Pulsed electric field (PEF) has been used for improving extraction of extra virgin olive oil (EVOO). However, the biological changes induced by the consumption of pulsed electric field-obtained extra virgin olive oil (PEFEVOO) have not been studied yet.

**Materials and methods:**

EVOO oils from Empeltre variety were prepared by standard (STD) cold pressure method involving crushing of the olives, malaxation and decanting and by this procedure including an additional step of PEF treatment. Chemical analyses of EVOO oils were done. Male and female *Apoe*-deficient mice received diets differing in both EVOOs for 12 weeks, and their plasma, aortas and livers were analyzed.

**Results:**

PEF application resulted in a 17% increase in the oil yield and minimal changes in chemical composition regarding phytosterols, phenolic compounds and microRNA. Only in females mice consuming PEF EVOO, a decreased plasma total cholesterol was observed, without significant changes in atherosclerosis and liver steatosis.

**Conclusion:**

PEF technology applied to EVOO extraction maintains the EVOO quality and improves the oil yield. The equivalent biological effects in atherosclerosis and fatty liver disease of PEF-obtained EVOO further support its safe use as a food.

## Introduction

Several epidemiological studies have linked Mediterranean diet to low rates of metabolic diseases and prolonged longevity (1–3). Although there are some geographical modifications in this dietary pattern, virgin olive oil (VOO) is always the main source of fat (4). Interventions using VOO as part of a Mediterranean diet have resulted in decreased mortality in primary (5) and secondary cardiovascular (6) preventions. Among the wide spectrum of benefits attributed to virgin olive oil consumption are also included positive modulation of lipid metabolism, insulin resistance, immune-inflammatory pathways, antithrombotic effect, detoxification of reactive species, improving endothelial dysfunction and blood pressure control (4, 7). Virgin olive oil or its highest quality preparation (extra-virgin olive oil, EVOO) is composed of an oily matrix of monounsaturated fatty acid-containing triglycerides and a minor fraction dubbed unsaponifiable (8). The latter represents about 0.5–1.5% of oil, is composed of hydrocarbons, triterpenes, phytosterols and phenolic compounds and is considered responsible for the VOO biological effects (4, 8–10).

Given the relevance of the unsaponifiable fraction, several procedures have been proposed to increase the recovery of these components in VOO. In this regard, high-speed centrifugation of olive paste produced an oil enriched in phytosterols, tocopherols, triterpenes and waxes that showed a great anti-atherosclerotic potential (11). High power ultrasound treatment of olive paste increased the green sensorial attribute (12). High vacuum conditions during the malaxation of Picual VOO resulted in an increased oleacein content (13).

Identification of food-derived microRNAs (miRNAs) in animal and plant kingdoms opened the possibility of host gene regulation when ingested (14–16). Despite their instability when unprotected, dietary miRNAs can be found encapsulated in extracellular vesicles, which contributes to reduce their degradation (17). Although recent evidence suggest that dietary miRNAs could be used as marker of food production (i.e., milk) system (18), the presence of food-derived miRNAs from *Olea europea* has been poorly described (19). A recent review addressing the current controversies regarding plant miRNA and trans-kingdom transference can be found in Saiyed et al. (20).

Pulsed electric field (PEF) is a non-thermal process characterized by the electroporation of cell membranes which is used for improving extraction of intracellular compounds of interest from plant-based foods (21, 22). PEF technology applied to EVOO extraction from Arbequina, Arroniz, Carolea, Coratina, and Ottobratica varieties slightly improved the extraction efficiency, without affecting its physic-chemical characteristics (23–26). However, in none of these works, the possible biological effects of its consumption have been addressed. The present work was set up to characterize the EVOO prepared using PEF from Empeltre variety from chemistry and biological perspectives. The former will analyze composition of fatty acids, squalene, triterpenes, phenols, phytosterols and microRNAs, as a novel food parameter. The second aspect will be explored by feeding Western diets containing standard or PEF EVOO and analyzing the atherosclerosis and fatty liver disease in *Apoe*-deficient mice, a spontaneous model of both pathologies (27, 28).

## Materials and methods

### Olive fruit

The study was conducted with olive fruits of the Empeltre variety from groves located in Zaragoza (Aragón, Spain). Olive fruits were harvested on the first days of December 2018 and transported to the laboratory for olive oil extraction. Maturation indexes ranged from 0 (intense green skin) to 7 (black skin and 100% purple flesh) as proposed by Hermoso et al. (29).

### Oil extraction system

Both STD and PEF EVOO samples were obtained by processing a continuous flow of 80 kg of olives per h in an Oliomio 80 plus machine (MORI-TEM s.r.l., Barberino Tavarnelle FI, Italy) equipped with a knife crusher, a horizontal continuous malaxer (working at room-temperature) and a two-phase decanter. In the PEF procedure, a collinear chamber of 2 cm between the electrodes and an inner diameter of 2 cm was added between the crusher and the malaxer to process the ground olives. In total, 600 kg of olives were processed, 300 kg for each procedure. In total with yields of 10–12%, around 60 l of oil were obtained. For chemical analysis, the oils were filtered under vacuum using Whatman^®^ paper no. 42 (Merck, Darmstadt, Germany).

Oil yields were analyzed as described by Martínez et al. (30). Basically, 2 kg of the olive pastes obtained from the malaxer at different time periods (0–60 min) were centrifuged at 1,370 × *g* for 2 min at 21 ± 1°C in an Abencor lab System (MC2 System, Seville, Spain), then the oil was collected and weighted. The oil extraction yield was calculated as the percentage of olive oil extracted from the olive paste.

### Pulsed electric field treatments

After milling, the olive paste was PEF treated using a PM1-10 generator (Energy Pulse Systems LDA, Lisbon, Portugal). A high-voltage probe (Tektronix, P6015A, Wilsonville, OR, USA) and an oscilloscope (Tektronix, TBS 1102B-EDU) were used to record and measure the shape and amplitude of the pulse. The PEF treatment involved 12 monopolar square-wave pulses of 20 μs at an electric field strength of 2 kV/cm (3.9 kJ/kg). The temperature of the olive paste was registered before and during the PEF treatments. The initial temperature of the mass was around 20°C and never surpassed 22°C in any of the assayed conditions.

### Extra virgin olive oil analysis

The chemical analyses of both virgin olive oils were carried at the analytical unit of the Instituto de la Grasa (CSIC, Seville, Spain) using the official methods proposed by the European Commission (31). In addition, an olive oil panel tested the obtained virgin olive oils to establish their category as EVOO.

### Isolation of microRNAs from extra virgin olive oil samples

To isolate RNAs from EVOO, 165 ml of EVOO were mixed with 20 ml of water and homogenized with TissueRuptor (Qiagen, Madrid, Spain) during 5 min at 4°C. After that, samples were centrifuged during 10 min at 10,000 × *g*. Oleum phase was discarded and then, another 165 ml of EVOO were incorporated to repeat the process. Small RNAs were extracted from the aqueous phase using QIAzol Lysis Reagent (Qiagen) according to the manufacturer’s instructions. DNase digestion was also performed. After eluting the RNA of each sample in 50 μl RNase-free water, the RNA quality and integrity was evaluated using Agilent 2100 Bioanalyzer (Agilent Technologies, Santa Clara, CA, USA).

### Small ribonucleic acid sequencing and data analysis

Library preparation and sequencing were carried out at “Fundación Parque Científico de Madrid” (Madrid, Spain). Briefly, total RNA was used as input for library preparation, using the NEBNext^®^ Multiplex Small RNA Library Prep Set for Illumina^®^ (New England BioLabs Ipswich, MA, USA) following the manufacturer’s instructions. Equimolecular pools of small RNA regions (< 200 nt) were purified by polyacrylamide gel electrophoresis. The library pool was denatured and seeded on a NextSeq v2.5 flowcell (Illumina, San Diego, CA, USA) and sequenced using a NextSeq 500 High Output kit v2.5 (Illumina) in a 1 × 75 single-read sequencing run on a NextSeq 500 sequencer (Illumina). In order to generate clean data, the initial sequences were purged from low-quality reads, repeated and adaptor sequences. Bowtie2 was used for alignment against the reference miRNA database from *Olea europea* L (32). Feature counting was carried out using HTSeq-count. Before differential expression analysis, the obtained count matrix was normalized using the TMM method available within the edgeR library from the R coding environment. The complete data set is available from https://www.ncbi.nlm.nih.gov/sra/PRJNA894959.

### Animals and experimental procedure

*Apoe*-deficient mice on C57BL/6J genetic background were obtained from Charles River (Charles River Laboratories, Barcelona, Spain) and bred at the animal care facility located in the *Centro de Investigación Biomédica de Aragón*. To establish groups with similar initial weight and plasma cholesterol, 2-month-old mice (28 males and 32 females), were weighed, blood samples taken from the facial vein (after 4-h fasting) and their cholesterol analyzed. Four groups of *Apoe*-deficient mice were allocated, 2 groups for males and another 2 for females, and housed in sterile filter-top cages in rooms maintained under a 12-h light/12-h dark cycle. All had *ad libitum* access to food and water. Mouse experiments were carried out in accordance with the EU Directive 2010/63 on the protection of animals used for scientific purposes and the study protocol was approved by the Ethics Committee for Animal Research of the University of Zaragoza (PI61/18).

The dietary intervention lasted 12 weeks, where solid intakes were monitored weekly and body weights every 2 weeks. At the end of the experiment, food was withdrawn for 4 h, and the mice were weighed and then sacrificed by suffocation in a CO_2_ chamber. Blood samples were drawn by cardiac puncture, and plasma and serum were centrifuged at 3,000 × *g* for 10 min. The livers were removed, frozen in liquid nitrogen and stored at –80°C until processing and an aliquot was stored in buffered formaldehyde. Heart and aorta were perfused with PBS, hearts were filled with OCT Tissue-Tek^®^ (Sakura Finetek, Barcelona, Spain), frozen in liquid nitrogen and stored at –80°C, while dissected aortas were kept in buffered 10% formaldehyde at 4°C.

### Diets

During the intervention, mice received a Western-style purified diet containing 20% of standard extra-virgin olive oil (STD EVOO) or pulse electric field-obtained extra-virgin olive oil (PEF EVOO) and 0.15% cholesterol. These diets differing in the type of fat, STD EVOO and PEF EVOO, were prepared following the recommendations of the Nutrient Requirements of Laboratory Animals (33) and their components were previously described (28). After preparing the diets, they were frozen, lyophilized and immediately stored at –20°C in vacuum bags until used.

### Plasma parameters

Total plasma cholesterol and triglyceride concentrations were measured in a microtiter assay, using Infinity™ commercial kits (Thermo Scientific, Madrid, Spain), glucose (BioSystems, Barcelona, Spain) and non-esterified fatty acids (NEFA) (Fujifilm Wako chemicals, Richmond VA, USA) according to the manufacturer’s instructions. Total serum apolipoprotein A1 (APOA1) was quantified by ELISA (34) and arylesterase activity of paraoxonase (PON1) as previously described (35). Plasma lipoprotein profile was determined in 100 μl of pooled plasma samples from each group and sex by fast protein liquid chromatography (FPLC) gel filtration using a Superose 6B column (GE Healthcare, Chicago, Il, USA) as previously described (36). The presence of reactive oxygen species (ROS) was assessed by measuring the conversion of 2,7- dichlorofluorescein diacetate into fluorescent dichlorofluorescein (37) in FPLC-isolated fractions corresponding to the different lipoproteins (38), using equal amount of total cholesterol for each fraction.

### Evaluation of atherosclerotic lesions

*En face* analyses of dissected aortas, the cross-sectional analyses of aortic roots and aortic lesion characteristics were carried out as previously described (28).

### Hepatic histological analyses

Paraffin sections (4 μm) from the livers stored in formaldehyde were stained with hematoxylin and eosin, and a slide scanner Zeiss AsioScan.Z1 (Zeiss, Oberkochen, Germany) was used to capture all preparations. Lipid droplets were evaluated quantifying their areas in each liver section using Adobe Photoshop CS3 (Adobe Inc., San Jose, CA, USA) and expressed as percentage of total liver section as previously described (39).

### Ribonucleic acid extraction and quantification

Ribonucleic acid (RNA) from each liver was isolated using Tri reagent (Sigma). DNA contaminants were removed by TURBO DNAse treatment using the DNA removal kit from Thermo Fisher Scientific (Madrid, Spain). RNA was quantified by absorbance at 260 and 280 nm using a SPECTROstar^Nano^ and software version 2.12 (BGM LABTECH, Ortenberg, Germany). The A260/280 ratio was higher than 1.7 in all samples. The integrity of the 28S and 18S ribosomal RNAs was verified by 1% agarose gel electrophoresis. Ethidium-bromide stained gels were exposed to UV light and images were captured using a Geldoc Universal Hood II and the software Quantity One version 4.6.6 (BioRad, Madrid, Spain).

#### Reverse transcriptase and quantitative polymerase chain reaction (RT-qPCR)

DNA-free total RNA of each sample (500 ng) were reverse transcribed using the PrimeScript™ RT Reagent Kit (Takara Bio Inc., Kusatsu, Japan) according manufacturer instructions. The RT-qPCR reactions were done using PowerUp™ SYBR™ Green Master Mix reagents from Applied Biosystems (Bedford, USA) according to manufacturer instructions. All reactions were determined by duplicate using the 2^–ΔΔCt^ method and normalized the results using the gene *Rnu6*. The primers for oeu-miR-31 5P used were TTCCACGGCTTTCTTGAACTTC and the universal qPCR primer CCAGTGCAGGGTCCGAGGTA (Invitrogen, Madrid, Spain), and for *Rnu6* the direct sequence was CTCGCTTCGGCAGCACATA and the reverse sequence CGAATTTGCGTGTCATCCT.

### Statistical analyses

Data are shown as means ± SD. Variables not showing normal distribution (according to the Shapiro–Wilk’s test) or homology of variance were analyzed using the one-tailed Mann–Whitney’s *U* test. The Statistical Package for Social Sciences version 15 (SPSS, Chicago, IL, USA) and Prism 5 software for Windows (GraphPad, S. Diego, CA, USA) were used for statistical analyses.

## Results

### Olive oil yield and chemical analyses

**FIGURE 1:**
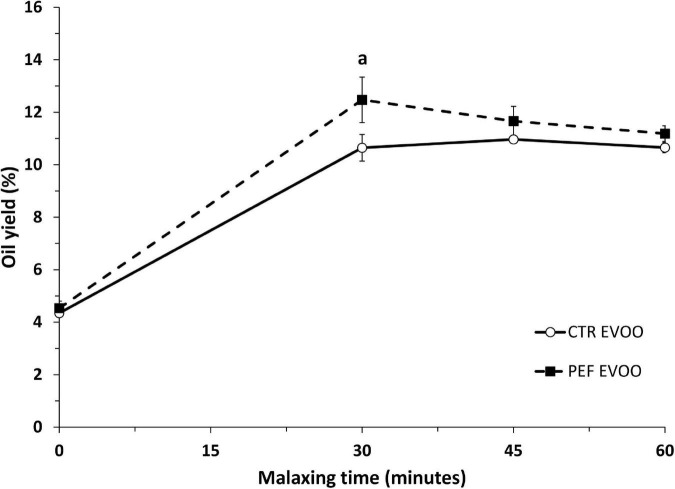
Extravirgin olive oil (EVOO) yields according to different procedures. Data were obtained using Abencor method of the EVOO at 21 ± 1°C at different malaxing times. Data are means ± SD for each point of triplicate determinations. Statistical analysis was carried out by Student’s *t*-test. *^a^p* < 0.05.

The maturation index of the Empeltre olives varied between 6 and 7 with a 100% of black skin. Figure 1 shows the olive oil yield obtained at different malaxing times for STD and PEF samples. As observed for PEF samples, 30 min after malaxing, maxima yields were already obtained. At that time point, an extra and significant yield of 1.9 ± 0.3% was obtained for PEF samples, which represents a 17% increase yield using PEF compared to STD extraction. These yield differences were reduced and without statistical significant by prolonging the malaxing time.

The complete set of chemical analyses is shown in Supplementary Tables 1 and 2 and both oils meet all the analytical requirements to be qualified as EVOO according to the normative that marks the Commission Regulation 2568/91 of 11 July 1991, based on their physicochemical quality (acidity ≤ 0.8% oleic acid; peroxide value ≤ 20 meq O_2_/kg; K270 ≤ 0.22; K232 ≤ 2.50). The main differences between both EVOOs (Table 1) were the peroxide index (6.9 and 8.2 meq O_2_/kg of fat), total sterols (1,263 and 1,372 mg/kg) and total phenolic compounds (115 and 121 mg/kg of tyrosol for STD and PEF EVOO, respectively). As reflected in Table 1 showing microRNA composition of EVOO, twelve miRNAs were characterized and present in the oils. Out of them, oeu-miR-31-5p was significantly higher in PEF and was not detected in STD EVOO (Supplementary Table 2).

**TABLE 1:** Main differences in chemical composition of used EVOO.

	STD EVOO	PEF EVOO
Peroxide index (mEq O_2_/kg)	6.9	8.2
K_232_	1.59	1.77
K_270_	0.07	0.09
ΔK	<0.01	<0.01
Rancimat at 110°C (hours)	12	11.3
Free fatty acids (% oleic acid)	0.11	0.14
Saturated fatty acids (%)	14.56	14.57
Monounsaturated fatty acids (%)	75.9	75.93
Polyunsaturated fatty acids (%)	9.54	9.5
Unsaponifiable (%)	1.27	1.26
Erythrodiol and uvaol (mg/kg)	11,000	12,000
Squalene (mg/kg)	4,708	4,708
Total sterols (mg/kg)	1,263	1,372
Aldehydic aglycon ligstroside (mg/kg of tyrosol)	5	10
Total phenolic compounds (mg/kg of tyrosol)	115	121
Total secoiridoid derivatives (mg/kg of tyrosol)	68	71
Tyrosol (mg/kg of tyrosol)	4	6
Hydroxytyrosol acetate (mg/kg of tyrosol)	2	3
Dialdehydic aglycon decarboxymethyl ligstroside (mg/kg of tyrosol)	42	41
**microRNAs (read counts)**		
oeu_mir_31_5p	0	6
oeu_miR166g	6,186	10,849
oeu_miR166q	267	318
oeu_mir_29_5p	5	10
oeu_miR168a	258	382
oeu_miR156b	11	9
oeu_mir_34_5p	2	2
oeu_mir_13_5p	49.5	58
oeu_mir_37_5p	1	1
oeu_mir_10_5p	8	10
oeu_mir_16_3p	10,135	14,122
oeu_miR156k	1	1

### Somatometric and plasma parameters of mice

During the 12 weeks of dietary intervention, the experimental groups did not show statistical differences in body weight gains and feed consumption in both sexes consuming Western diets containing STD or PEF EVOO, as shown in Supplementary Figure 1. The analysis of plasma parameters only showed statistical difference in female total cholesterol, without significant changes in triglycerides, glucose, NEFA, APOA1, and paraoxonase 1 activity from both sexes (Table 2). The observed differences in plasma total cholesterol in females lead us to study the composition of the lipoproteins in the experimental groups.

**TABLE 2:** Plasma parameters.

	Standard EVOO	PEF EVOO
**Males**		
Triglycerides (mg/dl)	206 ± 69	199 ± 62
Total cholesterol (mg/dl)	505 ± 101	523 ± 65
Glucose (mg/dl)	264 ± 91	257 ± 74
NEFA (mg/dl)	118 ± 37	110 ± 34
APOA1 (arbitrary units)	1.31 ± 0.22	1.18 ± 0.3
PON1 (IU/L)	50,862 ± 8,321	53,514 ± 12,105
VLDL ROS (arbitrary units)	7,801 ± 527	6,012 ± 247
LDL ROS (arbitrary units)	14,128 ± 1,058	3,853 ± 486^A^
HDL ROS (arbitrary units)	7,169 ± 207	2,996 ± 4,639
HDL + LDL ROS (arbitrary units)	11,384 ± 4,259	4,639 ± 989
**Females**		
Triglycerides (mg/dl)	193 ± 50	189 ± 36
Total cholesterol (mg/dl)	588 ± 68	498 ± 47^A^
Glucose (mg/dl)	250 ± 61	249 ± 60
NEFA (mg/dl)	106 ± 23	104 ± 17
APOA1 (arbitrary units)	0.66 ± 0.21	0.58 ± 0.15
PON1 (IU/L)	42,347 ± 7,094	46,631 ± 11,295
VLDL ROS (arbitrary units)	13,032 ± 245	18,931 ± 595^A^
LDL ROS (arbitrary units)	13,734 ± 777	16,982 ± 659
HDL ROS (arbitrary units)	14,627 ± 807	16,840 ± 1,080
HDL + LDL ROS (arbitrary units)	16,826 ± 715	17,575 ± 548

Data are individual values with their means ± SD for each group. Statistical analysis was carried out using Student’s t-test. ^A^*P* < 0.001 vs. standard EVOO.

### Plasma lipoprotein characterization

The analysis of the composition of lipoproteins separated by FPLC showed small differences in female total and esterified cholesterol as previously observed in plasma total cholesterol (Table 1), being slightly lower in the PEF EVOO group (Supplementary Figures 2B,D). In males, no differences in total and esterified cholesterol between both EVOOs were observed (Supplementary Figures 2A,C). There were minimal differences in lipoprotein APOA1 with a higher peak in HDL from both sexes in mice fed the PEF EVOO-containing diet (Supplementary Figures 2E,F).

The ROS contents in the different lipoproteins are shown in Table 2. The males consuming the STD EVOO-containing diet presented a higher ROS level in all analyzed lipoproteins, being statistically significant the increase in LDL. In contrast, the results in females were the opposite, those consuming the PEF EVOO-containing diet showed a discrete increase that only reached statistical significance in VLDL fraction.

### Hepatic ribonucleic acid expression using RT-PCR

The presence of oeu-miR-31-5p was searched in the livers of mice fed the PEF EVOO-containing diet. No such message of oeu-miR-31-5p was detected in both groups of mouse livers using the standard RT-qPCR procedure or conventional RT-PCR and PCR products resolved into a 2% agarose gel stained with ethidium bromide (data not shown). However, *Rnu6*, a classical reference gene expression for small RNA studies was detected with either procedure (data not shown).

### Evaluation of aortic lesions

**FIGURE 2:**
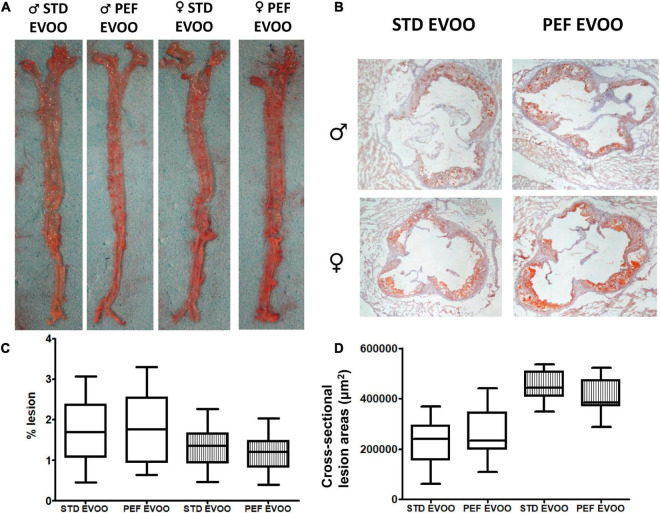
Effect of EVOO extraction procedure on atherosclerotic lesions in *Apoe*-KO mice after the dietary intervention. Panel **(A)** depicts representative *en face* aorta images and panel **(B)**, cross-sectional aortic sections. Panel **(C)** represents results from *en face* analyses expressed as % of lesion, and panel **(D)** the cross-sectional lesion areas in μm^2^. Results are shown as 5–95 percentiles for each group using white boxes for males and striped boxes for females. Statistical analysis was carried out by Mann–Whitney’s-*U* test.

**FIGURE 3:**
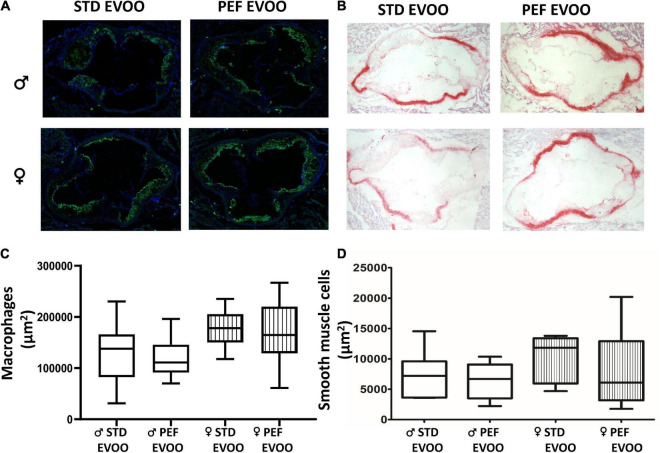
Effect of EVOO extraction procedure on atherosclerotic plaque characteristics in *Apoe*-KO mice after the dietary intervention. Representative aortic images corresponding to immunofluorescence detection of CD68 macrophages **(A)** and α-ACTIN detection of smooth muscle cells **(B)**. Quantification of macrophages as CD68 staining area (μm^2^) **(C)** and α-ACTIN surface staining as μm^2^ **(D)**. Results are shown as 5–95 percentiles for each group using empty boxes for males and striped boxes for females. Statistical analysis was carried out by Mann–Whitney’s-*U* test.

The presence of atherosclerotic foci throughout the entire aortic tree was evaluated by *en face* analysis (Figure 2A). Simultaneously, growth of atherosclerotic plaques was assessed by cross-sectional sections of the aortic roots (Figure 2B). In any of the procedures, no significant differences between the two used diets were observed in both sexes (Figures 2C,D). In order to characterize quality of atherosclerotic plaques, immunostaining of macrophages (Figure 3A) and smooth muscle cells (SMC) (Figure 3B) was carried out. Quantitative evaluation of those results resulted in no significant changes in the areas occupied by macrophages, according to CD68 staining, between both EVOO oils in both sexes (Figure 3C). Likewise, the presence of smooth muscle cells, evidenced by α-ACTIN immunohistochemistry, did not show statistically significant changes when administered the different diets in both sexes (Figure 3D).

### Hepatic histological analyses

**FIGURE 4:**
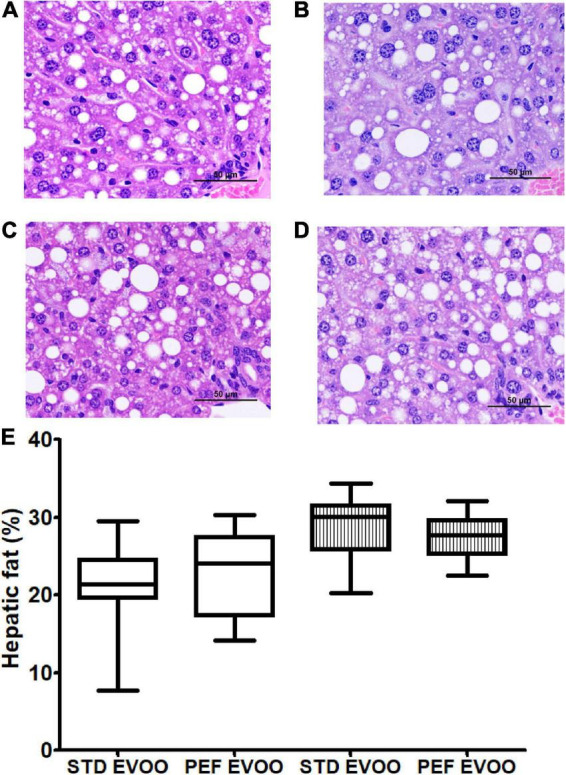
Hepatic histological analyses of *Apoe*-KO mice fed with the different diets. Representative liver micrographs from male mice consuming standard EVOO **(A)** and PEF EVOO **(B)**. Female mice consuming standard EVOO **(C)** and PEF EVOO **(D)**. Liver sections from each animal were stained with hematoxylin and eosin and evaluated blindly. Morphometric evaluation of lipid droplet area **(E)**. Results are shown as 5–95 percentiles for each group. Statistical analysis was carried out by Mann–Whitney’s-*U* test.

In order to test the influence of both diets on the development of fatty liver disease, hepatic histological analyses were carried out. Representative micrographs from males consuming standard (Figure 4A) and PEF EVOO (Figure 4B) and from female mice consuming standard (Figure 4C) and PEF EVOO (Figure 4D) are shown. A quantitative morphometric approach of lipid droplet areas is displayed in panel E of Figure 4. No significant changes were observed by using the different diets in both sexes.

## Discussion and conclusion

The present investigation was carried out to study the influence of the PEF technology on the EVOO extraction and its properties from Empeltre variety. This procedure improved the oil yield, and reduced the malaxing time maintaining similar quality of EVOO (Figure 1 and Table 1). A slight increase in phytosterols, phenolic compounds and oeu-miR-31-5p were observed in the PEF-prepared EVOO. Using *Apoe*-deficient mice as an animal model that develops atherosclerosis and liver steatosis, the biological properties of EVOO obtained by PEF technology on these pathologies and their plasma surrogate markers were studied.

Our results showed that the PEF procedure using Empeltre olives increased the oil yield in a 17% with 30 min of malaxing time respect the conventional EVOO extraction. These results are in agreement with those obtained using the Abencor method in Arbequina variety and ever higher than the 7% increase found by Guderjam et al. (23). However, these yields are slightly lower than those reported (20%) using industrial oil mills with the PEF technology (25). Another important aspect, according to our results (Figure 1), was the reduction of the malaxing time to 30 min to obtain such increased yield. Once again, our results reinforce the obtained by Abenoza et al. in Arbequina cultivar at 15°C (24). At these temperatures (15–21°C), no off-flavor or bad taste associated with PEF treatment was detected (22). Overall, the incorporation of PEF for the olive oil extraction industry may decrease its production costs by using reduced malaxing times and temperatures and providing increased yields without compromising current standards of EVOO quality.

The slight increase in phytosterols and phenolic compounds (Table 1 and Supplementary Table 1) observed in PEF-prepared Empeltre EVOO was a constant observed in other studies using this technology (22). Phytosterols are compounds located mainly inside the cell membranes (40), and the increase in the amount of total sterols in the PEF EVOO vs. STD EVOO is suggesting that the PEF breaks more membranes and cell structures than the standard procedure. Further support for this statement is provided by the increased phenolic compounds and oeu-miR-31 5p observed in the PEF EVOO. The variation of the peroxide index from 6.9 to 8.2 (mEq O_2_/kg), could be caused by the endogenous oxidases of the olive paste which remain more time in contact with the oil in the PEF EVOO, due to a greater breakdown of cellular structures. However, the values of free fatty acids of 0.11 and 0.14 for standard and PEF-EVOO were notoriously lower than those reported for this variety (0.61 ± 0.04) obtained by the Abencor method (41). The minimal differences in free fatty acids and peroxide index further reinforce the quality of the PEF EVOO from a chemical point of view.

In order to explore the biological effects of the PEF vs. STD EVOO, *Apoe*-deficient mice as an animal model that develops liver steatosis and atherosclerosis, received Western diets containing either of the oils, and their plasma, livers and arterial trees were analyzed. In plasma, the only significant difference was a lower total cholesterol concentration in female mice fed the PEF EVOO (Table 2), a result confirmed by analyzing the cholesterol distribution in plasma lipoproteins using FPLC (Supplementary Figures 2B,D). This effect could be attributed to the higher amount of phytosterols present in this diet and the reported hypocholesterolemic effect of these compounds (42). Interestingly, this effect was sex-specific, so were the oxidative properties of lipoproteins. In this regard, lipoproteins prepared from males consuming PEF EVOO were less oxidized than those obtained from standard EVOO and the opposite took place in females. Despite these changes in plasma lipoproteins, considered surrogate markers of atherosclerosis (43), no significant changes were observed in presence of atherosclerotic foci (Figures 2A,C), nor in lesion areas (Figures 2B,D). Likewise, the atherosclerotic plaque quality assessed by its macrophage and smooth muscle cell contents did not differ between these two EVOO (Figure 3). Finally, the hepatic lipid accumulation (Figure 4) did not show differences between both diets either. Globally, the studied biological properties of PEF EVOO support its equivalence to standard EVOO.

In conclusion, using Empeltre cultivar, our results show that the PEF applied to EVOO extraction improves the oil yield percentage, and reduces the malaxing time maintaining the same quality of EVOO. This represents an important advantage for olive oil industry of saving time and costs while obtaining a higher oil yield that might compensate the costs of introducing the PEF technology. The use of PEF technology was translated into an EVOO with slightly increased contents in phytosterols, total phenolic compounds and oeu-miR-31-5p which may be responsible for the reduction in total cholesterol particularly observed in female *Apoe* deficient mice, and a slight change in the oxidation profile of the different lipoproteins. No significant changes were observed on pathological outcomes such as atherosclerosis or development of fatty liver disease. These equivalent biological effects of PEF-obtained EVOO support its safe use as a food, in addition its increased yield will reduce the cost for consumers. The latter might further contribute to increase its consumption favoring a better adherence to Mediterranean diet and, in this way, a wholesome dietary pattern.

## Data availability statement

The data presented in this study are deposited in the https://www.ncbi.nlm.nih.gov/sra/repository, accession number: PRJNA894959.

## Ethics statement

This animal study was reviewed and approved by Ethics Committee for Animal Research of the University of Zaragoza (PI61/18).

## Author contributions

RM-B, IÁ-L, ACS-G, JR, JO, and MN designed research. RM-B, MR, TH-C, CB, AD, ML, IÁ-L, ACS-G, JR, CA, JCS, and MN conducted research. MR, TH-C, CB, and MN analyzed data. RM-B, IÁ-L, ML, JO, and MN wrote the manuscript. JO had primary responsibility for final content. All authors read and agreed to the published version of the manuscript.

## References

[1] Keys A Mienotti A Karvonen MJ Aravanis C Blackburn H Buzina R The diet and 15-year death rate in the seven countries study. *Am J Epidemiol.* (1986) 124:903–15. 3776973 10.1093/oxfordjournals.aje.a114480

[2] Trichopoulou A Costacou T Bamia C Trichopoulos D. Adherence to a Mediterranean diet and survival in a Greek population. *N Engl J Med.* (2003) 348:2599–608. 12826634 10.1056/NEJMoa025039

[3] Guasch-Ferré M Willett WC. The Mediterranean diet and health: a comprehensive overview. *J Intern Med.* (2021) 290:549–66. 10.1111/joim.13333 34423871

[4] Gaforio JJ Visioli F Alarcón-de-la-Lastra C Castañer O Delgado-Rodríguez M Fitó M Virgin olive oil and health: summary of the III international conference on virgin olive oil and health consensus report, JAEN (Spain) 2018. *Nutrients.* (2019) 11:2039. 10.3390/nu11092039 31480506 PMC6770785

[5] Estruch R Ros E Salas-Salvadó J Covas M-I Corella D Arós F Primary prevention of cardiovascular disease with a Mediterranean diet supplemented with extra-virgin olive oil or nuts. *N Engl J Med.* (2018) 378:e34. 10.1056/NEJMoa1800389 29897866

[6] Delgado-Lista J Alcala-Diaz JF Torres-Peña JD Quintana-Navarro GM Fuentes F Garcia-Rios A Long-term secondary prevention of cardiovascular disease with a Mediterranean diet and a low-fat diet (CORDIOPREV): a randomised controlled trial. *Lancet.* (2022) 399:1876–85. 10.1016/S0140-6736(22)00122-2 35525255

[7] Guasch-Ferré M Li Y Willett WC Qi S Sampson L Salas-Salvadó J Consumption of olive oil and risk of total and cause-specific mortality among U.S. adults. *J Am Coll Cardiol.* (2022) 79:101–12. 10.1016/j.jacc.2021.10.041 35027106 PMC8851878

[8] Lou-Bonafonte JM Arnal C Navarro MA Osada J. Efficacy of bioactive compounds from extra virgin olive oil to modulate atherosclerosis development. *Mol Nutr Food Res.* (2012) 56:1043–57. 10.1002/mnfr.201100668 22760979

[9] Markellos C Ourailidou ME Gavriatopoulou M Halvatsiotis P Sergentanis TN Psaltopoulou T. Olive oil intake and cancer risk: a systematic review and meta-analysis. *PLoS One.* (2022) 17:e0261649. 10.1371/journal.pone.0261649 35015763 PMC8751986

[10] Del Saz-Lara A Lopez de Las Hazas MC Visioli F Davalos A. Nutri-epigenetic effects of phenolic compounds from extra virgin olive oil: a systematic review. *Adv Nutr.* (2022) 13:2039–60. 10.1093/advances/nmac067 35679085 PMC9526845

[11] Acin S Navarro MA Perona JS Arbones-Mainar JM Surra JC Guzman MA Olive oil preparation determines the atherosclerotic protection in apolipoprotein E knockout mice. *J Nutr Biochem.* (2007) 18:418–24. 10.1016/j.jnutbio.2006.08.005 17049830

[12] Bejaoui MA Sánchez-Ortiz A Aguilera MP Ruiz-Moreno MJ Sánchez S Jiménez A High power ultrasound frequency for olive paste conditioning: effect on the virgin olive oil bioactive compounds and sensorial characteristics. *Innov Food Sci Emerg Technol.* (2018) 47:136–45.

[13] Veneziani G Selvaggini R Taticchi A Urbani S Esposto S Servili M. High vacuum applied during malaxation in oil industrial plant: influence on virgin olive oil extractability and quality. *Innov Food Sci Emerg Technol.* (2022) 79:103036.

[14] Zhang L Hou D Chen X Li D Zhu L Zhang Y Exogenous plant MIR168a specifically targets mammalian LDLRAP1: evidence of cross-kingdom regulation by microRNA. *Cell Res.* (2012) 22:107–26. 10.1038/cr.2011.158 21931358 PMC3351925

[15] Chin AR Fong MY Somlo G Wu J Swiderski P Wu X Cross-kingdom inhibition of breast cancer growth by plant miR159. *Cell Res.* (2016) 26:217–28. 10.1038/cr.2016.13 26794868 PMC4746606

[16] Del Pozo-Acebo L Lopez de Las Hazas MC Margolles A Davalos A Garcia-Ruiz A. Eating microRNAs: pharmacological opportunities for cross-kingdom regulation and implications in host gene and gut microbiota modulation. *Br J Pharmacol.* (2021) 178:2218–45. 10.1111/bph.15421 33644849

[17] Del Pozo-Acebo L Lopez de Las Hazas MC Tome-Carneiro J Del Saz-Lara A Gil-Zamorano J Balaguer L Therapeutic potential of broccoli-derived extracellular vesicles as nanocarriers of exogenous miRNAs. *Pharmacol Res.* (2022) 185:106472. 10.1016/j.phrs.2022.106472 36182038

[18] Abou El Qassim L Le Guillou S Royo LJ. Variation of miRNA content in cow raw milk depending on the dairy production system. *Int J Mol Sci.* (2022) 23:11681. 10.3390/ijms231911681 36232984 PMC9569736

[19] Mico V Martin R Lasuncion MA Ordovas JM Daimiel L. Unsuccessful detection of plant MicroRNAs in beer, extra virgin olive oil and human plasma after an acute ingestion of extra virgin olive oil. *Plant Foods Hum Nutr.* (2016) 71:102–8. 10.1007/s11130-016-0534-9 26872816

[20] Saiyed AN Vasavada AR Johar SRK. Recent trends in miRNA therapeutics and the application of plant miRNA for prevention and treatment of human diseases. *Future J Pharm Sci.* (2022) 8:24. 10.1186/s43094-022-00413-9 35382490 PMC8972743

[21] Heinz V Raso J. *Pulsed Electric Fields Technology for the Food Industry: Fundamentals and Applications.* Berlin: Springer (2006). p. 1–245.

[22] Raso J Heinz V Alvárez I Toepfl S. *Pulsed Electric Fields Technology for the Food Industry: Fundamentals and Applications.* 2nd ed. Berlin: Springer (2022). p. 1–561.

[23] Guderjan M Topfl S Angersbach A Knorr D. Impact of pulsed electric field treatment on the recovery and quality of plant oils. *J Food Eng.* (2005) 67:281–7.

[24] Abenoza M Benito M Saldana G Alvarez I Raso J Sanchez-Gimeno AC. Effects of pulsed electric field on yield extraction and quality of olive oil. *Food Bioprocess Technol.* (2013) 6:1367–73.

[25] Puertolas E de Maranon IM. Olive oil pilot-production assisted by pulsed electric field: impact on extraction yield, chemical parameters and sensory properties. *Food Chem.* (2015) 167:497–502. 10.1016/j.foodchem.2014.07.029 25149017

[26] Veneziani G Esposto S Taticchi A Selvaggini R Sordini B Lorefice A Extra-virgin olive oil extracted using pulsed electric field technology: cultivar impact on oil yield and quality. *Front Nutr.* (2019) 6:134. 10.3389/fnut.2019.00134 PMC6737034 31555654

[27] Guillen N Acin S Navarro MA Surra J Arnal C Lou-Bonafonte JM Knowledge of the biological actions of extra virgin olive oil gained from mice lacking apolipoprotein E. *Rev Esp Cardiol.* (2009) 62:294–304. 10.1016/s1885-5857(09)71560-9 19268075

[28] Martinez-Beamonte R Sanchez-Marco J Lazaro G Barco M Herrero-Continente T Serrano-Megias M Dietary avian proteins are comparable to soybean proteins on the atherosclerosis development and fatty liver disease in apoe-deficient mice. *Nutrients.* (2021) 13:1838. 10.3390/nu13061838 34072167 PMC8227708

[29] Hermoso M Uceda M García A Morales B Frías ML Fernández A. *Elaboración del Aceite de Oliva de Calidad.* Sevilla: Consejería de Agricultura y Pesca (1991). p. 5–92.

[30] Martínez JM Muñoz E Alba J Lanzón A. Informe sobre la utilización del analizador de rendimientos “abencor”. *Grasas Aceites.* (1975) 26:379–85.

[31] European Commission [EC]. Commission regulation (EEC) No 2568/91 of 11 July 1991 on the characteristics of olive oil and olive-residue oil and on the relevant methods of analysis. *Off J Eur Comm.* (1991) 34:L248.

[32] Yanik H Turktas M Dundar E Hernandez P Dorado G Unver T. Genome-wide identification of alternate bearing-associated microRNAs (miRNAs) in olive (*Olea europaea* L.). *BMC Plant Biol.* (2013) 13:10. 10.1186/1471-2229-13-10 23320600 PMC3564680

[33] Reeves PG Rossow KL Lindlauf J. Development and testing of the ain-93 purified diets for rodents – results on growth, kidney calcification and bone mineralization in rats and mice. *J Nutr.* (1993) 123:1923–31. 10.1093/jn/123.11.1923 8229309

[34] Navarro MA Carpintero R Acin S Arbones-Mainar JM Calleja L Carnicer R Immune-regulation of the apolipoprotein A-I/C-III/A-IV gene cluster in experimental inflammation. *Cytokine.* (2005) 31:52–63. 15878672 10.1016/j.cyto.2005.03.002

[35] Gabas-Rivera C Barranquero C Martinez-Beamonte R Navarro MA Surra JC Osada J. Dietary squalene increases high density lipoprotein-cholesterol and paraoxonase 1 and decreases oxidative stress in mice. *PLoS One.* (2014) 9:e104224. 10.1371/journal.pone.0104224 25117703 PMC4130590

[36] Martinez-Beamonte R Navarro MA Acin S Guillen N Barranquero C Arnal C Postprandial changes in high density lipoproteins in rats subjected to gavage administration of virgin olive oil. *PLoS One.* (2013) 8:e55231. 10.1371/journal.pone.0055231 23383120 PMC3558467

[37] Navab M Hama SY Hough GP Subbanagounder G Reddy ST Fogelman AM. A cell-free assay for detecting HDL that is dysfunctional in preventing the formation of or inactivating oxidized phospholipids. *J Lipid Res.* (2001) 42:1308–17. 11483633

[38] Arbones-Mainar JM Navarro MA Guzman MA Arnal C Surra JC Acin S Selective effect of conjugated linoleic acid isomers on atherosclerotic lesion development in apolipoprotein E knockout mice. *Atherosclerosis.* (2006) 189:318–27. 10.1016/j.atherosclerosis.2006.01.015 16530768

[39] Guillen N Acin S Navarro MA Perona JS Arbones-Mainar JM Arnal C Squalene in a sex-dependent manner modulates atherosclerotic lesion which correlates with hepatic fat content in apoE-knockout male mice. *Atherosclerosis.* (2008) 197:72–83. 10.1016/j.atherosclerosis.2007.08.008 17854812

[40] Barba FJ Esteve MJ Frigola A. Bioactive components from leaf vegetable products. *Stud Nat Prod Chem.* (2014) 41:321–46.

[41] Rey-Giménez R Sánchez-Gimeno AC. Authenticity in olive oils from an empeltre clonal selection in Aragon (Spain): how environmental, agronomic, and genetic factors affect sterol composition. *Foods.* (2022) 11:2587. 36076773 10.3390/foods11172587 PMC9455585

[42] Gylling H Plat J Turley S Ginsberg HN Ellegård L Jessup W Plant sterols and plant stanols in the management of dyslipidaemia and prevention of cardiovascular disease. *Atherosclerosis.* (2014) 232:346–60. 24468148 10.1016/j.atherosclerosis.2013.11.043

[43] Duivenvoorden R de Groot E Stroes ES Kastelein JJ. Surrogate markers in clinical trials – challenges and opportunities. *Atherosclerosis.* (2009) 206:8–16. 19327774 10.1016/j.atherosclerosis.2008.12.009

