# Supplementary material for: Pulsed electric field increases the extraction yield of extra virgin olive oil without loss of its biological properties
Source: Front Nutr. 2022 Nov 22;9:1065543. doi: 10.3389/fnut.2022.1065543 (PMC9722962; doi:10.3389/fnut.2022.1065543)

Supplementary Table 1. Detailed chemical composition of used EVOO.

|  | STD EVOO | PEF EVOO |
| --- | --- | --- |
| Peroxide index (mEq O_2_/ kg) | 6.9 | 8.2 |
| K232 | 1.59 | 1.77 |
| K270 | 0.07 | 0.09 |
| ΔK | <0.01 | <0.01 |
| Rancimat at 110 ºC (hours) | 12 | 11.3 |
| Fatty acid ethyl esters (mg/ kg) | 3 | <5 |
| Fatty acid alkyl esters (mg/ kg) | 4 | 5 |
| Polymerized triglycerides (%) | <0.5 | <0.5 |
| Free fatty acids (% oleic acid) | 0.11 | 0.14 |
| C-14:0 (miristic) % | 0.01 | 0.01 |
| C-16:0 (palmitic) % | 12.35 | 12.36 |
| C-16:1 (palmitoleic) % | 1.21 | 1.2 |
| C-17:0 (margaric) % | 0.09 | 0.09 |
| C-17:1 (margaroleic) % | 0.24 | 0.24 |
| C-18:0 (stearic) % | 1.6 | 1.6 |
| C-18:1 (oleic) % | 74.08 | 74.13 |
| C-18:2 (linoleic) % | 8.86 | 8.82 |
| C-20:0 (araquidonic) % | 0.34 | 0.33 |
| C-18:3 (linolenic) % | 0.69 | 0.68 |
| C-20:1 (eicosenoic) % | 0.37 | 0.37 |
| C-22:0 (behenic) % | 0.11 | 0.11 |
| C-24:0 (lignoceric) % | 0.06 | 0.06 |
| Trans oleic (t-C18:1) % | 0.01 | 0.01 |
| Trans linoleic + trans linolenic (t-C18:2 + t-C18:3) % | 0.02 | 0.03 |
| Saturated fatty acids % | 14.56 | 14.57 |
| Monounsaturated fatty acids % | 75.9 | 75.93 |
| Polyunsaturated fatty acids % | 9.54 | 9.5 |
| Unsaponificable (%) | 1.27 | 1.26 |
| Erythrodiol and uvaol (mg/ kg) | 11000 | 12000 |
| Oleanolic acid (mg/ kg) | 15 | 16 |
| Ursolic acid (mg/ kg) | <2 | <2 |
| Maslinic acid (mg/ kg) | 21 | 23 |
| Squalene (mg/ kg) | 4708 | 4708 |
| β-carotens (mg/ kg) | 2 | 2 |
| Feofitins (mg/ kg) | <5 | <5 |
| Total sterols (mg/ kg) | 1263 | 1372 |
| Cholesterol (%) | <0.1 | <0.1 |
| Brassicasterol (%) | <0.1 | <0.1 |
| Campesterol (%) | 2.8 | 2.8 |
| Stigmasterol (%) | 0.6 | 0.6 |
| D^7^-stigmastenol (%) | 0.4 | 0.5 |
| β-sitosterol (%) | 95.2 | 95.3 |
| Aliphatic alcohols (C22+C24+C26+C28) mg/ kg | 129 | 124 |
| Aliphatic waxes (C40+C42+C44+C46) mg/ kg | 125 | 127 |
| Aliphatic waxes (C42+C44+C46) mg/ kg | 84 | 85 |
| Total phenolic compounds (mg/ kg of tyrosol) | 115 | 121 |
| Total orthodiphenols (mg/ kg of tyrosol) | 26 | 26 |
| Total secoiridoid derivatives (mg/ kg of tyrosol) | 68 | 71 |
| Hydroxytyrosol (mg/ kg of tyrosol) | 1 | 1 |
| Tyrosol (mg/ kg of tyrosol) | 4 | 6 |
| Vanillic acid (mg/ kg of tyrosol) | 0 | 0 |
| Vanillin (mg/ kg of tyrosol) | 0 | 0 |
| P-cumaric acid (mg/ kg of tyrosol) | 0 | 0 |
| Hydroxytyrosol acetate (mg/ kg of tyrosol) | 2 | 3 |
| Dialdehydic decarboxymethyl oleuropein aglycone (mg/ kg of tyrosol) | 7 | 6 |
| Tyrosol acetate (mg/ kg of tyrosol) | 2 | 4 |
| Dialdehydic aglycon decarboxymethyl ligstroside (mg/ kg of tyrosol) | 42 | 41 |
| Pinoresinol (mg/ kg of tyrosol) | 1 | 1 |
| Cinnamic acid (mg/ kg of tyrosol) | 0 | 0 |
| 1-acetoxypinoresinol (mg/ kg of tyrosol) | 33 | 32 |
| Aldehydic aglycon oleuropein (mg/ kg of tyrosol) | 14 | 15 |
| Aldehydic aglycon ligstroside (mg/ kg of tyrosol) | 5 | 10 |
| Ferulic acid (mg/ kg of tyrosol) | 0 | 0 |
| Luteolin (mg/ kg of tyrosol) | 3 | 2 |
| Apinegin (mg/ kg of tyrosol) | 0 | 0 |
| α-tocopherol (mg/ kg) | 148 | 148 |
| β-tocopherol (mg/ kg) | <2 | <2 |
| γ-tocopherol (mg/ kg) | 14 | 14 |
| δ-tocopherol (mg/ kg) | <2 | <2 |

Supplementary Table 2. Detected microRNA composition of used EVOO.

|  | STD EVOO 1 | STD EVOO 2 | PEF EVOO 1 | PEF EVOO 2 | Log FC | Log CPM | P Value | FDR |
| --- | --- | --- | --- | --- | --- | --- | --- | --- |
| oeu_mir_31_5p | 0 | 0 | 8 | 4 | 5.42792 | 7.86356 | 0.00099 | 0.01190 |
| oeu_miR166g | 4813 | 7559 | 13323 | 8376 | 0.40783 | 18.59733 | 0.20769 | 1.00000 |
| oeu_miR166q | 196 | 339 | 295 | 342 | -0.14830 | 13.77058 | 0.48676 | 1.00000 |
| oeu_mir_29_5p | 4 | 6 | 8 | 12 | 0.57231 | 8.79988 | 0.60035 | 1.00000 |
| oeu_miR168a | 226 | 291 | 330 | 434 | 0.12055 | 13.89796 | 0.65032 | 1.00000 |
| oeu_miR156b | 3 | 19 | 14 | 4 | -0.53726 | 9.13546 | 0.71611 | 1.00000 |
| oeu_mir_34_5p | 3 | 1 | 2 | 2 | -0.44330 | 7.57826 | 0.76244 | 1.00000 |
| oeu_mir_13_5p | 28 | 71 | 43 | 73 | -0.12808 | 11.34386 | 0.83350 | 1.00000 |
| oeu_mir_37_5p | 2 | 0 | 1 | 0 | -1.46195 | 7.03789 | 0.86867 | 1.00000 |
| oeu_mir_10_5p | 2 | 13 | 5 | 15 | 0.11741 | 8.96239 | 0.93123 | 1.00000 |
| oeu_mir_16_3p | 9603 | 10668 | 16036 | 12209 | 0.01701 | 19.15551 | 0.96136 | 1.00000 |
| oeu_miR156k | 0 | 2 | 2 | 0 | -0.22684 | 7.14471 | 1.00000 | 1.00000 |

**Supplemental Figure 1.** **Body weight and feed consumption**. Graphs correspond to body weight follow-up during diet intervention in males (A) and females (B) and feed consumption of males (C) and females (D). Data are means ± SD for each group. Statistical analysis was carried out by Student’s t test.

Females

Males


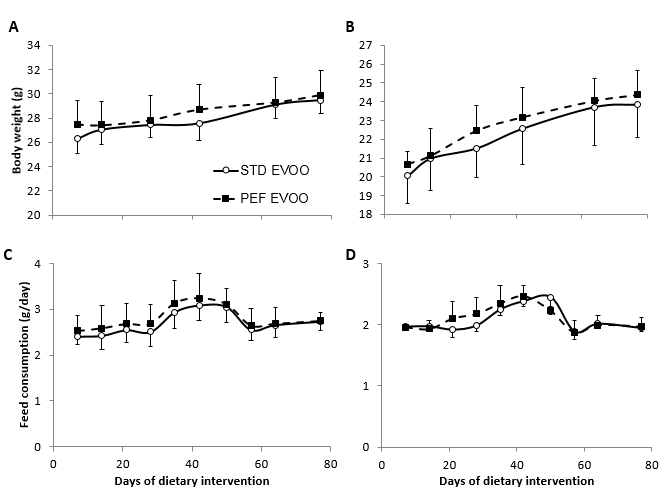


**Supplemental Figure 2: Representative lipoprotein profiles of mice consuming both EVOO for 12 weeks.** FPLC-collected fractions analyzed for total cholesterol in males (A), and females (B), esterified cholesterol in males (C) and females (D), and APOA1 in males (E) and females (F). Fractions 14 to 18 correspond to VLDL, 19 to 27 to LDL, 28 to 33 to cholesterol-rich HDL and 34 to 37 to cholesterol-poor HDL.


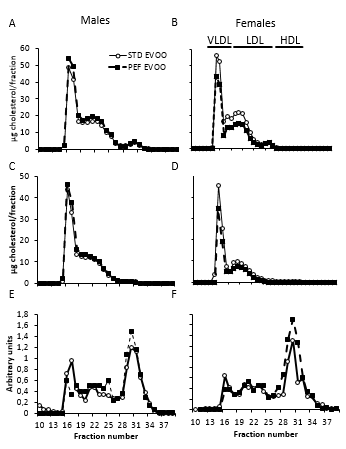

Supplement: Supplementary file 1 [file Data_Sheet_1.docx]
